# Identification of cellular senescence-specific genes by comparative transcriptomics

**DOI:** 10.1038/srep31758

**Published:** 2016-08-22

**Authors:** Taiki Nagano, Masayuki Nakano, Akio Nakashima, Kengo Onishi, Shunsuke Yamao, Masato Enari, Ushio Kikkawa, Shinji Kamada

**Affiliations:** 1 Biosignal Research Center, Kobe University, 1-1 Rokkodai-cho, Nada-ku, Kobe 657-8501, Japan; 2 Department of Biology, Graduate School of Science, Kobe University, 1-1 Rokkodai-cho, Nada-ku, Kobe 657-8501, Japan; 3 Department of Bioresource Science, Graduate School of Agricultural Science, Kobe University, 1-1 Rokkodai-cho, Nada-ku, Kobe 657-8501, Japan; 4 Division of Refractory and Advanced Cancer, National Cancer Center Research Institute, 5-1-1 Tsukiji, Chuo-ku, Tokyo 104-0045, Japan

## Abstract

Cellular senescence is defined as permanent cell cycle arrest induced by various stresses. Although the p53 transcriptional activity is essential for senescence induction, the downstream genes that are crucial for senescence remain unsolved. Here, by using a developed experimental system in which cellular senescence or apoptosis is induced preferentially by altering concentration of etoposide, a DNA-damaging drug, we compared gene expression profiles of senescent and apoptotic cells by microarray analysis. Subtraction of the expression profile of apoptotic cells identified 20 genes upregulated specifically in senescent cells. Furthermore, 6 out of 20 genes showed p53-dependent upregulation by comparing gene expression between p53-proficient and -deficient cells. These 6 genes were also upregulated during replicative senescence of normal human diploid fibroblasts, suggesting that upregulation of these genes is a general phenomenon in senescence. Among these genes, 2 genes (*PRODH* and *DAO*) were found to be directly regulated by p53, and ectopic expression of 4 genes (*PRODH, DAO, EPN3*, and *GPR172B*) affected senescence phenotypes induced by etoposide treatment. Collectively, our results identified several proteins as novel downstream effectors of p53-mediated senescence and provided new clues for further research on the complex signalling networks underlying the induction and maintenance of senescence.

Cellular senescence was first described as a process of irreversible cell cycle arrest resulting from the limited replicative capacity of human diploid fibroblasts in culture^1^. Although this process, now termed as replicative senescence, is associated with telomere shortening^2^, senescence can be also induced independent of telomere erosion as a consequence of various forms of stress, such as activated oncogenes, reactive oxygen species (ROS), and DNA damage^3^,^4^,^5^, and is referred to as premature senescence. It has been suggested that DNA damage is a common mediator for various forms of senescence induced by diverse stimuli including telomere shortening^6^,^7^,^8^,^9^. In response to DNA damage, the DNA damage response (DDR) is initiated by phosphatidylinositol 3-kinase like kinases such as ATM and ATR. Once activated, ATM and ATR phosphorylate downstream checkpoint kinases Chk1 and Chk2, which in turn phosphorylate and activate p53 to affect cell cycle progression. The p53 tumor suppressor protein is a critical transcription factor that regulates cell proliferation and death^10^. Besides inducing senescence, activated p53 also modulates transient cell cycle arrest and apoptosis to act as a genome guardian. These cellular responses depend on the expression of respective sets of particular p53 target genes^11^,^12^.

Microarray-based gene expression profiling is frequently used to identify targets of transcription factors, and the list of p53 downstream effectors that mediate tumor suppressor function of p53 through induction of transient cell cycle arrest, senescence, and apoptosis is sizable^12^,^13^,^14^. However, the gene expression analyses concerning the different modes of p53 activation have identified overlapping sets of downstream p53 targets, and the p53 transcriptional targets required specifically for initiation and maintenance of senescence phenotypes have not been fully elucidated^15^,^16^. Although the Cdk inhibitor p21, a target of p53, has been considered as central to arrest cell cycle in senescent cells^17^,^18^, senescence induction cannot be explained by the function of p21 alone^19^,^20^.

In the present study, we developed an experimental system in which either senescence or apoptosis is preferentially induced in the same cell line by altering dosage of a DNA-damaging drug and compared gene expression profiles using microarray analysis in an attempt to distinguish genes specific for senescence from those that universally respond to DNA damage. Furthermore, we provide evidence that some of the identified genes are functionally involved in the senescence program.

## Results

### Senescence is induced by the low dose of etoposide, whereas apoptosis is triggered at higher doses

**Figure 1:**
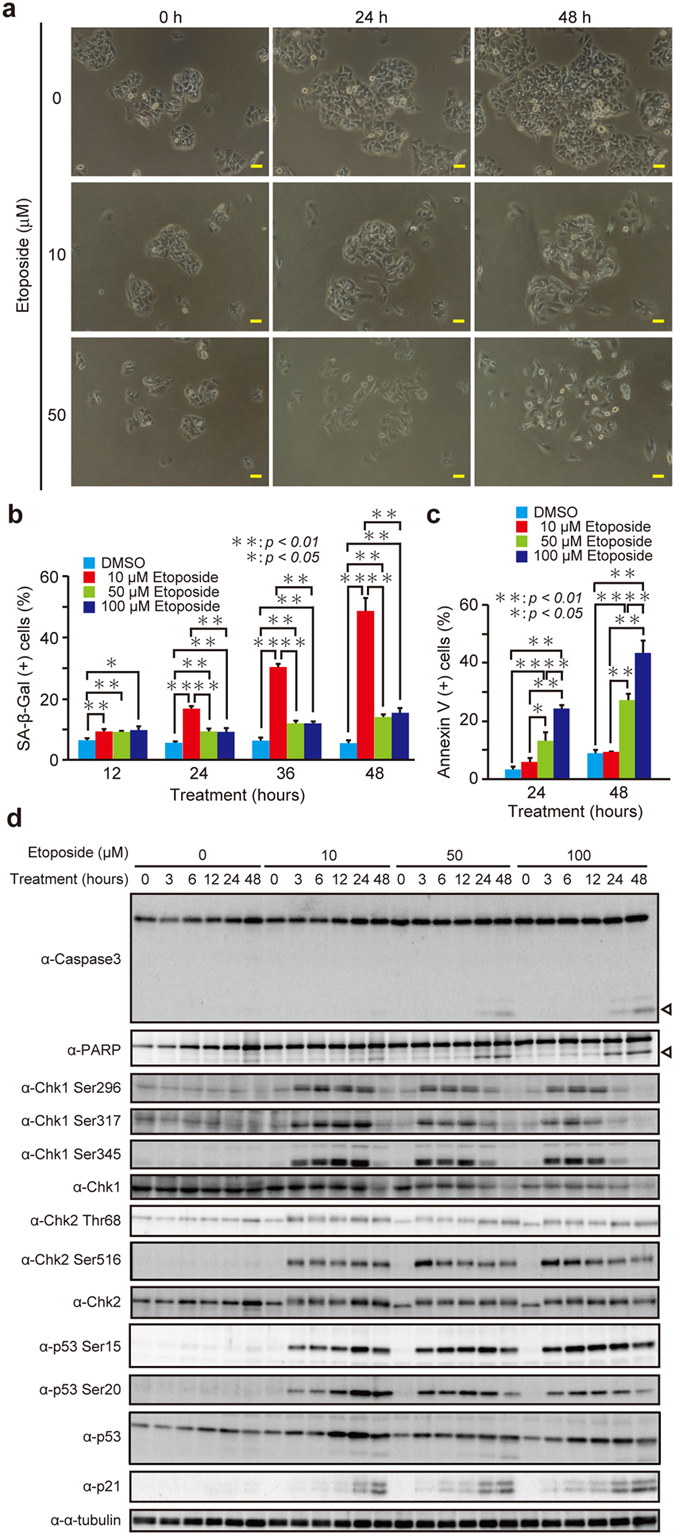
Senescence is induced by the low dose of etoposide, whereas apoptosis is triggered at higher doses in HepG2 cells. (**a**) HepG2 cells were treated with 10 and 50 μM etoposide for 24 and 48 h, and observed under microscope. Bars, 50 μm. (**b–d**) HepG2 cells treated with 10, 50, and 100 μM etoposide for indicated times were subjected to SA-β-Gal staining (**b**), Annexin V staining (**c**), or immunoblot analysis (**d**). Each immunoblot is representative of at least two independent experiments. Arrowheads indicate the cleaved form of the respective proteins. The uncropped images are shown in Supplementary Fig. 5. Data are mean ± SD. Statistical significance is shown using the Student’s *t*-test analysis (*n* = 3); *P < 0.05; **P < 0.01.

To develop an experimental system in which either senescence or apoptosis is specifically induced, we first investigated the phenotypes of two human tumor lines expressing the wild-type p53, hepatocarcinoma HepG2 cells and osteosarcoma U2OS cells, as a function of the dosage of etoposide, an anticancer drug that causes DNA double-strand breaks. After treatment of HepG2 cells with a relatively lower dose of etoposide (10 μM), we observed the cells showing the senescence-like growth arrest (Fig. 1a). In contrast, treatment with a higher dose of etoposide (50 μM) caused loss of overall cell shape, which represents the morphological sign of apoptosis. To evaluate the extent of senescence and apoptosis induced by different doses of etoposide, cells treated with etoposide at 10, 50, and 100 μM were examined for the markers of senescence and apoptosis. When the cells were treated with the low dose of etoposide (10 μM), the activity of senescence-associated β-galactosidase (SA-β-Gal), a well-known late senescence marker^21^, gradually increased in a time-dependent manner (Fig. 1b and Supplementary Fig. 1a). To assess apoptosis, cells treated with a range of etoposide doses were stained with Annexin V, as a marker of apoptosis (Fig. 1c). Approximately 10% of apoptotic cells were observed in cultures treated with the low dose of etoposide for 48 h, whereas about 30 and 40% of apoptotic cells were measured in cultures exposed to 50 and 100 μM etoposide, respectively. Consistent with this, immunoblot analysis revealed that cleavage of caspase-3 and poly(ADP-ribose) polymerase (PARP), a substrate for caspases, was detected at high etoposide doses (50 and 100 μM) but not at the low dose (10 μM) (Fig. 1d). These results suggest that etoposide at the low dose mainly induces senescence, whereas it causes primarily apoptosis at high doses in HepG2 cells.

To confirm these results, U2OS cells were treated with different doses of etoposide and bleomycin, another DNA damage-inducing drug that has a different mode of action from etoposide to induce DNA double-strand breaks^22^. Consistent with our findings in HepG2 cells, a low dose (2 μM) of etoposide and bleomycin reduced the colony-forming ability of U2OS cells (Supplementary Fig. 1b) and increased the number of SA-β-Gal-positive cells in a time-dependent manner (Supplementary Fig. 1c). In addition, apoptosis occurred at the high dose of etoposide (100 μM) but not at the low dose (2 μM) as evidenced by Annexin V staining and PARP cleavage (Supplementary Fig. 1d,e). These results indicate that senescence is also induced mainly by the low dose of the drugs, and apoptosis predominates at the high dose in U2OS cells.

### p53 transcriptional activation between 24 h and 36 h after treatment with the low dose of etoposide is required for senescence execution

Senescence is mediated through the activation of DDR kinases^6^,^7^,^8^,^9^. Consistently, inhibition or depletion of DDR components (ATM, ATR, or Chk1) effectively prevented etoposide-induced senescence (Supplementary Fig. 2a–d). To further obtain information on DDR kinases responsible for senescence and apoptosis induced by etoposide, we assessed the phosphorylation states of Chk1 and Chk2 (Fig. 1d). ATM and ATR phosphorylate Chk1 at Ser317 and Ser345, which induces functionally essential autophosphorylation at Ser296^23^. The phosphorylation levels of Chk1 at Ser296, Ser317, and Ser345 increased and remained high even after 24 h treatment with the low dose of etoposide, whereas at high doses, Chk1 phosphorylation increased during first 12 h but decreased 24 h after treatment, raising the possibility that a relatively prolonged activation of Chk1 could lead the cells to senescence. ATM and ATR also phosphorylate Chk2 at Thr 68, which promotes dimerization of Chk2 and autophosphorylation at Thr383, Thr387, and Ser516^23^. In contrast to Chk1, the phosphorylation levels of Chk2 at Thr68 and Ser516 similarly increased at both low and high doses.

To confirm the response of Chk1 phosphorylation to the different doses of etoposide, we applied U2OS cells for the analysis (Supplementary Fig. 1e). The phosphorylation of Chk1 at Ser345 increased gradually until 48 h after treatment with the low dose of etoposide, while at the high dose, Chk1 phosphorylation sharply reduced 36 h after treatment. Moreover, although ectopic expression of wild-type Chk1 promoted etoposide-induced senescence, no such effect was observed by overexpressing Chk1 mutant S345A (Supplementary Fig. 2e). These results suggest that the prolonged Chk1 activation after treatment with the low dose of etoposide may be necessary for senescence induction.

Since Chk1 activates p53 by phosphorylation, and p53 is a critical factor for senescence induction, we analyzed the phosphorylation states of p53 (Fig. 1d). The phosphorylation of p53 occurs at several sites, such as at Ser15 and Ser20, and these phosphorylation events are important for p53 stabilization. The phosphorylation levels of p53 at Ser20 dramatically increased until 24 h after treatment with the low dose of etoposide accompanying the increase of p53 protein, and sustained for up to 48 h, which is consistent with the prolonged activation of Chk1 (Fig. 1d). However, high doses of etoposide did not cause such a marked increase of Ser20 phosphorylation or the protein level of p53 during the period of 24–48 h, when compared to the low dose. Meanwhile, p53 phosphorylation at Ser15 increased similarly at both low and high doses throughout the time course of treatment. These results suggest that p53 activation through the phosphorylation at particular site(s) between 24 and 48 h after etoposide treatment may be needed to induce senescence.

**Figure 2:**
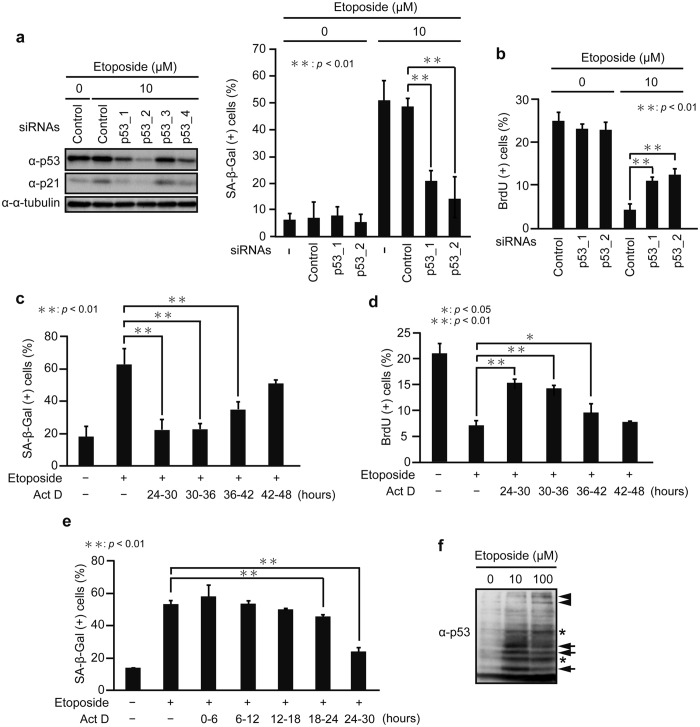
p53 transcriptional activation between 24 h and 36 h after treatment with the low dose of etoposide is required for senescence execution. (**a**) HepG2 cells transfected with siRNAs for *p53* (p53_1, p53_2, p53_3, and p53_4) were treated with 10 μM etoposide for 48 h. The expression levels of p53 and p21 were determined by immunoblot analysis (left), and the percentage of SA-β-Gal-positive cells was quantified (right). The uncropped images are shown in Supplementary Fig. 6a. (**b**) HepG2 cells treated as in (**a**) were subjected to BrdU incorporation assay, and the percentage of BrdU-positive cells was quantified. (**c,d**) HepG2 cells were treated with 10 μM etoposide for various times (24, 30, 36, and 42 h), and then Act D was added to the medium at a concentration of 50 ng/ml. After 6 h of incubation in the presence of Act D and etoposide, the drugs were washed out by replacing the medium, and the cells retreated only with etoposide up to for 48 h after initial exposure to etoposide were subjected to SA-β-Gal staining (**c**) and BrdU incorporation assay (**d**). (**e**) HepG2 cells cultured as in (**c**) but treated with Act D during 0–30 h after etoposide exposure as indicated were subjected to SA-β-Gal staining. (**f**) Lysates from HepG2 cells treated with 10 and 100 μM etoposide for 24 h were subjected to Phos-tag SDS-PAGE and immunoblotted with the anti-p53 antibody. Arrows indicate the bands showing stronger signals at 10 μM than 100 μM etoposide, and arrowheads depict the bands with stronger signals at 100 μM than 10 μM etoposide. Asterisks indicate the bands with no difference between low and high doses of etoposide. The uncropped images are shown in Supplementary Fig. 6b. Data are mean ± SD. Statistical significance is shown using the Student’s *t*-test analysis (*n* = 3); *P < 0.05; **P < 0.01.

To test whether etoposide-induced senescence is dependent on p53, we used siRNAs to deplete *p53*. Knockdown of *p53* remarkably decreased etoposide-induced senescence as determined by SA-β-Gal staining and 5-Bromo-2′-deoxyuridine (BrdU) incorporation assay (Fig. 2a,b and Supplementary Fig. 3), suggesting that senescence induced by the low dose of etoposide is highly dependent on p53. Next, to investigate whether senescence execution is dependent on p53 transcriptional activity between 24 and 48 h after treatment with the low dose of etoposide and, if so, determine at what time point transcription is required, the transcriptional inhibitor, actinomycin D (Act D), was added to the culture medium at four different time points (24, 30, 36, and 42 h) after exposure to etoposide. After 6 h of incubation in the presence of Act D and etoposide, the drugs were washed out by replacing the medium, and cells retreated only with etoposide up to for 48 h after initial etoposide exposure were subjected to SA-β-Gal staining and BrdU incorporation assay (Fig. 2c,d and Supplementary Fig. 4). When treated with Act D from 24 to 30 h (24–30 h) or 30–36 h after exposure to etoposide, senescence was markedly blocked, whereas inhibition of transcription 36–42 h after etoposide exposure partially suppressed senescence, and the addition of Act D after 42 h had no significant effect. These results suggest that the transcriptional activation of p53-target genes between 24 h and 36 h after etoposide treatment is required for senescence execution. Consistent with this idea, treatment with Act D during 0–6 h, 6–12 h, 12–18 h, and 18–24 h following etoposide exposure had little effect on SA-β-Gal activation (Fig. 2e).

Furthermore, we applied the Phos-tag SDS-polyacrylamide gel electrophoresis (SDS-PAGE) method to compare the overall p53 phosphorylation patterns after 24 h treatment with low and high doses of etoposide. The Phos-tag polymerized into the SDS-polyacrylamide gel binds to phosphate groups and enhances the phosphorylation-dependent mobility shift^24^,^25^. Lysates from HepG2 cells treated with low and high doses of etoposide for 24 h were subjected to Phos-tag SDS-PAGE. After treatment with etoposide, p53 was separated into several bands with large mobility differences, whereas no mobility shift was detected in the absence of etoposide (Fig. 2f). More importantly, the p53 banding patterns differed between low and high doses of etoposide. At the low dose, stronger signals were observed in the relatively lower bands, while more intense bands appeared at higher region at the high dose, reflecting the different phosphorylation patterns of p53 between low and high etoposide doses. Since some of the posttranslational modifications of p53, including phosphorylation, influence p53’s target gene selection^26^, these results further support the idea that distinct sets of target genes are transcriptionally activated by p53 in response to different doses of etoposide.

### Twenty genes are upregulated at only low but not high doses of etoposide, and *PRODH* and *DAO* are directly regulated by p53

To identify downstream transcriptional targets differentially expressed in cells treated with different doses of etoposide, we profiled the transcriptome of HepG2 cells treated with low and high doses of etoposide for 30 h using microarray analysis since this time point was the center of the time period in which senescence was most effectively inhibited by Act D (Fig. 2c–e and Supplementary Fig. 4). Gene expression profiling revealed that 126 genes were upregulated more than 3-fold at the low dose of etoposide as compared with control cells. In addition, when compared the expression profiles of these genes between low and high doses, 25 genes were found to be differentially upregulated by more than 2-fold at the low dose. After exclusion of 3 genes with well-established function in the processes, protein metabolism (*MDM2*), DNA replication (*PCNA*), and translesion DNA synthesis (*POLH*) with the purpose of identifying novel genes that function in senescence and of 2 genes encoding hypothetical proteins, 20 genes were selected for further analysis (Table 1). Genes previously shown to be involved in senescence induction, such as *E2F7, p21, BTG2*, and *SULF2*^17^,^18^,^27^,^28^,^29^,^30^, were identified to be upregulated in our screening, however, the expression of *p21, BTG2*, and *SULF2* increased to a similar extent at low and high doses (<2-fold difference). After confirming the microarray results by quantitative PCR (qPCR), 17 out of 20 selected genes showed higher expression at the low dose compared with that at the high dose (Table 1; right columns).

**Figure 3:**
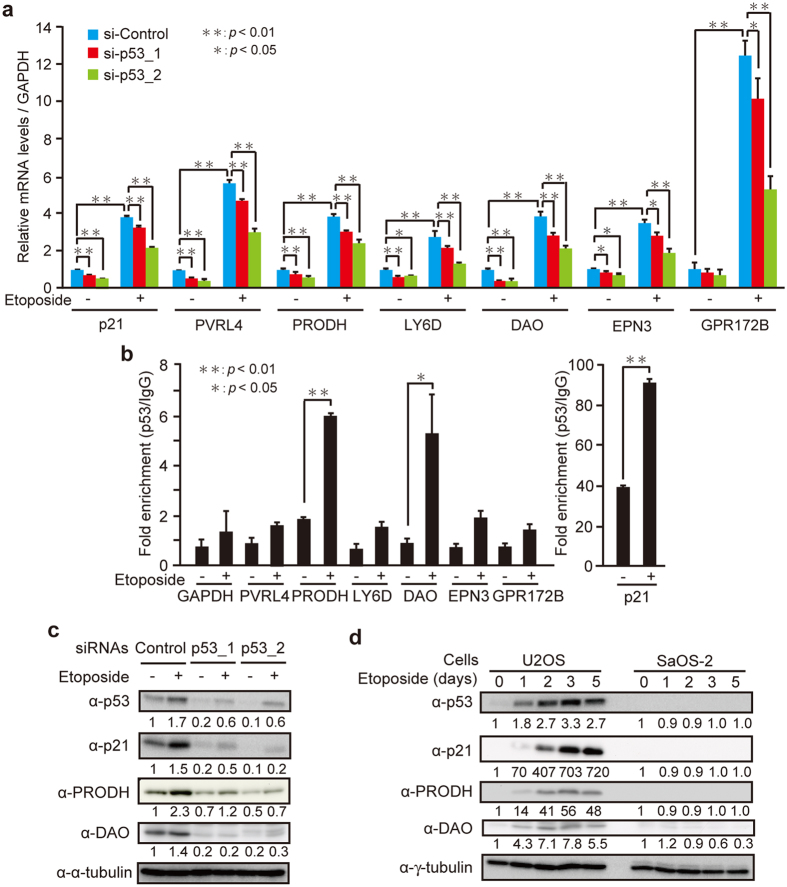
*PRODH* and *DAO* are identified as direct transcriptional targets of p53. (**a**) Relative mRNA levels of indicated genes in HepG2 cells transfected with *p53* siRNAs (p53_1 and p53_2) and treated with 10 μM etoposide for 30 h were determined by qPCR. (**b**) HepG2 cells treated with 10 μM etoposide for 30 h were subjected to ChIP assay with the anti-p53 antibody. The fold enrichment of p53 over normal IgG was determined by qPCR. (**c**) HepG2 cells were transfected with siRNAs for negative control (Control) and *p53* (p53_1 and p53_2). After incubation for 48 h, the cells were treated with 10 μM etoposide for 30 h, and the levels of indicated proteins were determined by immunoblot analysis. The protein levels relative to the α-tubulin level were quantified using NIH ImageJ software and are indicated at the bottom of each lane. The uncropped images are shown in Supplementary Fig. 7a. (**d**) U2OS and SaOS-2 cells were treated with 2 μM etoposide for 1, 2, 3, and 5 days, and the levels of indicated proteins were determined by immunoblot analysis. The protein levels relative to the γ-tubulin level were quantified as in (**c**). Protein levels at day 0 in U2OS and SaOS-2 cells were normalized to 1, respectively. The uncropped images are shown in Supplementary Fig. 7b. Data are mean ± SD. Statistical significance is shown using the Student’s *t*-test analysis (*n* = 3); *P < 0.05; **P < 0.01.

To investigate whether the transcriptional activation of the selected genes upon senescence is conserved in other cell lines, and whether it is dependent on p53, we compared U2OS and SaOS-2 cells, which have the wild-type p53 and are deficient of p53, respectively. qPCR analysis showed that 7 out of the 17 genes were upregulated by more than 2-fold in U2OS cells treated with etoposide and bleomycin (Table 2). In contrast, such increases were absent or reduced in SaOS-2 cells with the exception of the *IGFBP2* gene, suggesting that upregulation of 6 genes (*PVRL4, PRODH, LY6D, DAO, EPN3*, and *GPR172B*) upon etoposide treatment is dependent on p53 (Tables 2 and 3). Furthermore, these results were confirmed by using p53-knockdown HepG2 cells (Fig. 3a). Next, we tested whether p53 binds to the genomic regions of the 6 genes. By analyzing the sequences of these genes, we identified putative p53 response elements: in the promoters of *PVRL4, LY6D*, and *EPN3*; in the first intron of *DAO*; in the third intron of *PRODH* consistent with the previously reported p53-binding site^31^; in the fourth intron of *GPR172B*. Chromatin immunoprecipitation (ChIP) assay using the anti-p53 antibody in HepG2 cells revealed that p53 bound to the genomic regions of *PRODH* and *DAO* when treated with etoposide (Fig. 3b). Furthermore, the protein levels of PRODH and DAO also increased in response to etoposide in HepG2 and U2OS cells, which was abolished by *p53* depletion and was absent in SaOS-2 cells (Fig. 3c,d). These results suggest that PRODH and DAO are directly regulated by p53 upon DNA damage.

### The identified genes are induced during replicative senescence in Hs68 cells

**Figure 4:**
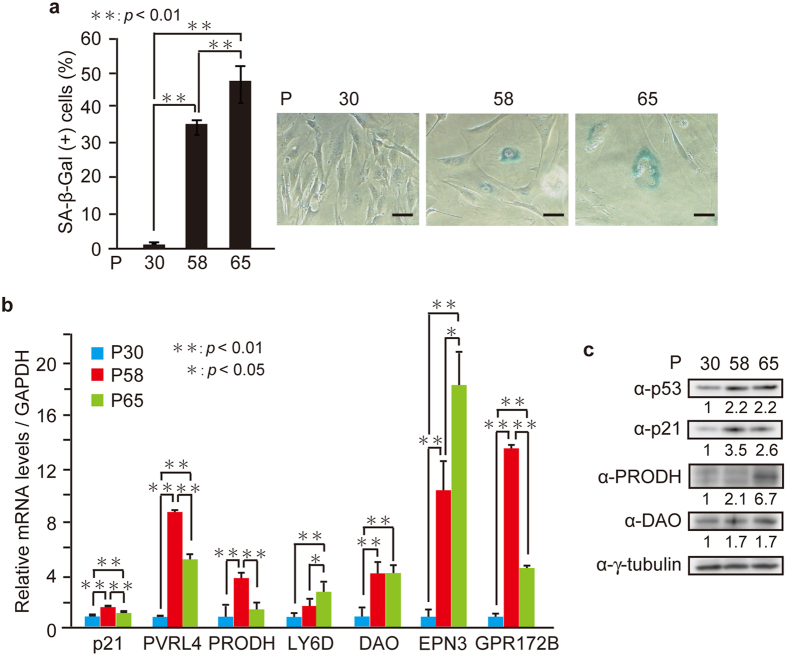
The identified genes are induced during replicative senescence in Hs68 cells. Hs68 cells at passage numbers 30, 58, and 65 (P30, P58, and P65) were subjected to SA-β-Gal staining (**a**), qPCR (**b**), and immunoblot analysis (**c**). (**a**) The percentage of SA-β-Gal-positive cells (left) and representative images (right) are shown. Bars, 50 μm. (**b**) Relative mRNA levels of indicated genes are shown. (**c**) The protein levels relative to the γ-tubulin level were quantified using NIH ImageJ software and are indicated at the bottom of each lane. The uncropped images are shown in Supplementary Fig. 8. Data are mean ± SD. Statistical significance is shown using the Student’s *t*-test analysis (*n* = 3); *P < 0.05; **P < 0.01.

To examine whether expression of the selected genes is induced during replicative senescence, we assessed the expression levels of the 6 genes in normal human diploid fibroblasts Hs68 cells triggered to undergo senescence by replicative exhaustion (Fig. 4). Induction of replicative senescence in aged Hs68 cells (at passages 58 and 65) was confirmed by SA-β-Gal staining (Fig. 4a). The mRNA levels of all 6 genes and the protein levels of PRODH and DAO were elevated during the culture passages to various extents (Fig. 4b,c). These results suggest that upregulation of these genes is a general phenomenon in senescence.

### PRODH, DAO, and EPN3 promote senescence, whereas GPR172B inhibits senescence

**Figure 5:**
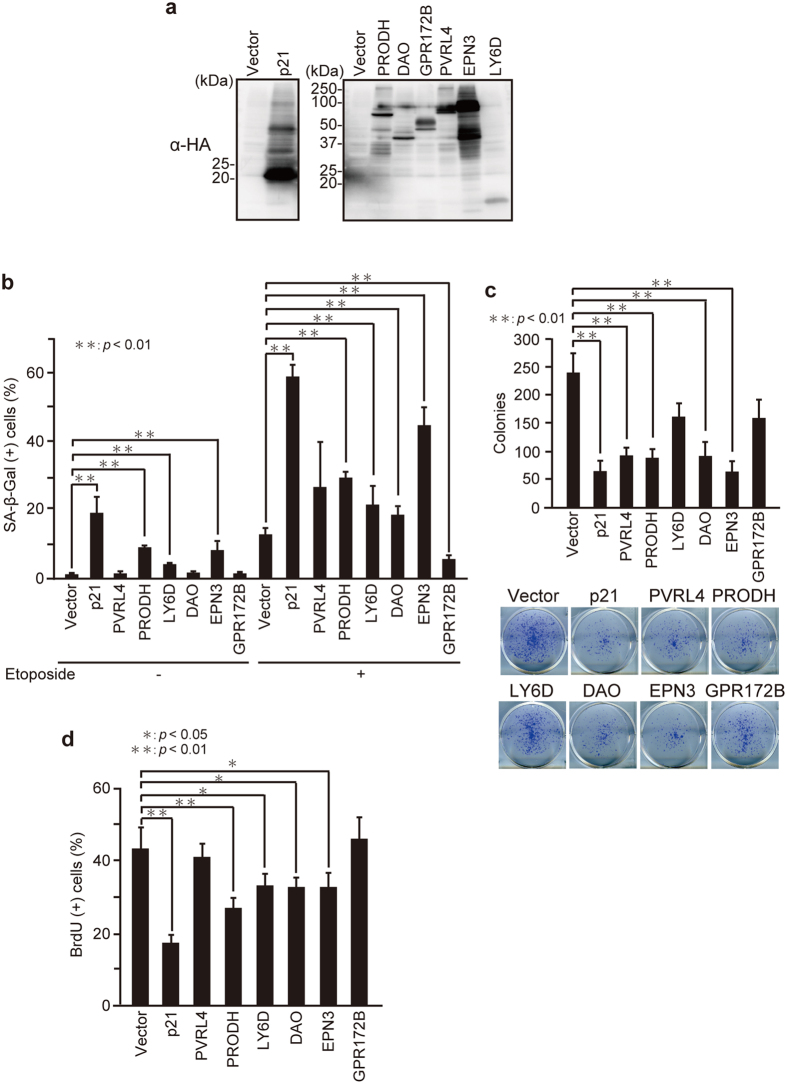
PRODH, DAO, and EPN3 promote senescence, whereas GPR172B inhibits senescence. (**a**) U2OS cells were transfected with pcDNA3-HA containing *p21, PRODH, DAO, GPR172B, PVRL4, EPN3*, and *LY6D*. After incubation for 24 h, lysates were subjected to immunoblot analysis with the anti-HA antibody. The uncropped image is shown in Supplementary Fig. 9. (**b,c**) U2OS cells overexpressed with p21, PVRL4, PRODH, LY6D, DAO, EPN3, and GPR172B, selected with G418, and treated with 2 μM etoposide were subjected to SA-β-Gal staining (**b**) and colony-formation assay (**c**). The number of colonies was counted using NIH ImageJ software. (**d**) Hs68 cells overexpressed with p21, PVRL4, PRODH, LY6D, DAO, EPN3, and GPR172B in combination with EGFP as a transfection marker for 24 h were subjected to BrdU incorporation assay. The percentage of BrdU-positive cells in GFP-expressing cells are shown. Data are mean ± SD. Statistical significance is shown using the Student’s *t*-test analysis (*n* = 3); *P < 0.05; **P < 0.01.

To test whether the 6 genes functionally contribute to the senescence program, expression vectors containing these genes were introduced individually into U2OS cells (Fig. 5a–c). Immunoblot analysis validated that each of the proteins was expressed in cells (Fig. 5a). Ectopic expression of p21 (positive control), PRODH, LY6D, and EPN3 enhanced senescence regardless of whether or not etoposide was present (Fig. 5b). Ectopic DAO facilitated etoposide-induced SA-β-Gal expression, whereas the cells overexpressing GPR172B showed decreased SA-β-Gal activity in the presence of etoposide. Furthermore, ectopic expression of p21, PRODH, DAO, PVRL4, and EPN3 decreased the colony-forming ability (Fig. 5c). In addition, Hs68 cells overexpressed with PRODH, LY6D, DAO, and EPN3 showed impaired proliferation as judged by BrdU incorporation assay (Fig. 5d). These results suggest that PRODH, DAO, and EPN3 can promote senescence, and that GPR172B may act to suppress senescence.

## Discussion

DNA-damaging anticancer drugs such as etoposide and bleomycin can induce both senescence and apoptosis^32^,^33^. Although this demonstrates that DNA damage is a critical initiator of senescence and apoptosis, the mechanism underlying the determination of the choice between senescence and apoptosis is currently unknown. The results presented in this study indicate that treatment of cells with relatively lower doses of etoposide induces senescence, whereas high doses of the drug induce apoptosis. In addition, we found that Chk1 activation is more prolonged in cells exposed to the low dose of etoposide, and that the transcriptional activity of p53 between 24 h and 36 h after etoposide treatment is required for senescence execution in HepG2 cells. In this time point, the phosphorylation patterns of p53 were differed between low and high doses of etoposide. Consistent with this, Chk1 has been reported to phosphorylate p53 at multiple sites within both N- and C-terminal regions and affect p53 transcriptional activity^34^,^35^. In conclusion, we reasoned that genes crucial for senescence execution were transcriptionally activated by p53 between 24 h and 36 h after etoposide treatment in HepG2 cells.

Comparison of gene expression profiles between cells treated with low and high doses of etoposide allowed us to identify 20 genes that were specifically upregulated in senescent cells. Among these genes, we confirmed that upregulation of 6 genes (*PVRL4, PRODH, LY6D, DAO, EPN3*, and *GPR172B*) were dependent on p53. To our knowledge, none of them has yet been linked to the senescence or ageing process. Furthermore, ChIP analysis validated the direct interaction of p53 with the genomic regions of *PRODH* and *DAO*. While *PRODH* has been shown to be directly regulated by p53^31^, the transcriptional activation of *DAO* by p53 has not been described. Recently, it has been reported that *DAO* expression is controlled by the binding of transcription factor Pax-5 to two *cis*-acting elements located 60 bp upstream (−60) and 4464 bp downstream (+4464) of the transcription start site of the *DAO* gene^36^. Since the putative p53-binding site targeted in our study (+2287) is distinct from those two elements, this result indicates that the DAO expression level may be cooperatively regulated by multiple transcription factors depending on the cell context.

We further found that overexpression of PRODH, DAO, and EPN3 promoted senescence. Among these proteins, PRODH and DAO are oxidases, both of which are known to produce ROS as byproducts of substrate oxidation^37^,^38^. It is widely accepted that ROS induce senescence thorough oxidative stress^7^,^8^,^9^,^39^. ROS levels increase in both replicative and premature senescence, and treatment of cells with a sublethal dose of H_2_O_2_ induces senescence^4^. Accordingly, ROS are considered to be strong candidates that mediate senescence-promoting effect of these oxidases. On the other hand, EPN3, a member of the epsin family, plays an important role as an accessory protein in clathrin-mediated endocytosis^40^. Endocytosis has been reported to negatively regulate senescence under normal growth conditions^41^, but at the same time clathrin-mediated endocytosis also promotes autophagy, a cellular catabolic process that contributes to senescence induction^42^,^43^,^44^, which leads us to predict that p53 utilizes multiple mechanisms to regulate senescence. Furthermore, we observed that ectopic expression of GPR172B inhibited senescence. This effect appears to be reasonable because GPR172B is a membrane transporter for riboflavin (also known as vitamin B2) that acts as a coenzyme for redox enzymes essential for cellular homeostasis^45^,^46^. This result raises the possibility that p53 also drives a negative feedback loop that limits activation of senescence-inducing signalling pathways to prevent accidental induction of senescence. However, further investigation is necessary to elucidate the precise mechanisms by which these genes influence the senescence program.

In conclusion, the comparative genome-wide expression analysis presented here elucidated novel genes specifically upregulated in senescence. In particular, the 6 genes are regulated by p53, and some of them are functionally involved in senescence possibly through multiple cellular processes, including oxidative stress response, endocytosis, and intracellular redox balancing system. These data on gene regulation by p53 thus enhance our understanding of the mechanism underlying p53-dependent senescence. Besides permanent growth arrest and SA-β-Gal, senescent cells show a wide variety of features, such as enlarged and flattened cell morphology, vacuole formation, inflammatory secretory phenotype, and heterochromatic foci formation; however, the detailed molecular mechanisms underlying these phenotypes are still unclear. Further functional characterization of the newly identified genes will hopefully lead to a better understanding of the molecular basis of senescence and its associated phenotypes.

## Methods

### Cell culture, treatment, and transfection

HepG2 (a human hepatocellular carcinoma line; a gift from Dr. S. Shimizu) cells were cultured in RPMI 1640 medium (Wako) supplemented with 10% fetal bovine serum (FBS). U2OS (a human osteosarcoma line; ATCC), SaOS-2 (a human osteosarcoma line; a gift from Dr. M. Takahashi), and Hs68 (normal human diploid fibroblasts; IFO50350, JCRB Cell Bank)^47^ cells were cultured in DMEM (Wako) supplemented with 10% FBS. For senescence induction, HepG2 cells were treated with 10 μM etoposide (Sigma Aldrich) for 48 h and used for subsequent assays, while U2OS cells were treated with etoposide or bleomycin (Wako) at 2 μM for 48 h and cultured in the medium without the drugs for additional 5 days to develop senescent phenotypes. CGK733 (Merck Millipore) or KU-55933 (Merck Millipore) was added to the medium 1 h before etoposide treatment. Inhibition of transcription was achieved by incubating the cells with 50 ng/ml Act D (Sigma Aldrich). Transfection with expression vectors was performed using FuGENE HD (Promega) according to the manufacturer’s instruction. The transfectants were selected with 800 μg/ml G418 (Wako) for 5 days where indicated.

### Senescence assays

For colony formation assay, 1000, 5000, or 10000 cells were plated in 35-mm dish, cultured for 7–10 days, and stained with crystal violet (Wako). Detection of SA-β-Gal activity was performed using Senescence β-Galactosidase staining kit (Cell Signaling Technology) according to the manufacturer’s instruction. Briefly, the cells were fixed with 2% formaldehyde/0.2% glutaraldehyde. After incubation with SA-β-Gal staining solution (1 mg/ml 5- bromo-4-chloro-3-indolyl-β-D-galactoside, 40 mM citric acid/sodium phosphate [pH 6.0], 5 mM potassium ferrocyanide, 5 mM potassium ferricyanide, 150 mM NaCl, 2 mM MgCl_2_) for 12 h (HepG2 cells) or 24 h (U2OS and Hs68 cells), the cells were examined under fluorescence microscope (model BZ-8000; Keyence). At least 100 cells were counted to determine the percentage of SA-β-Gal positive cells. For BrdU incorporation assay, cells were labeled with 10 μM BrdU (Sigma) for 6 h (HepG2 cells) or 24 h (Hs68 cells). For BrdU immunostaining, the labeled cells were fixed with 3.7% formaldehyde in PBS and permeabilized with 0.5% TritonX-100. DNA was hydrolyzed by exposing cells to 2 N HCl for 10 min, and then cells were incubated with anti-BrdU antibody (BD Pharmingen, 555627) in Can Get Signal immunostain Solution B (TOYOBO) overnight at 4 °C followed by incubation with TRITC-conjugated secondary antibodies (Molecular Probes, T2762) for 1 h at room temperature. After staining nuclei with 10 μM Hoechst 33342, cells were examined under fluorescence microscope (model BZ-9000; Keyence).

### Apoptosis assay

The Annexin-V-FLUOS Staining Kit (Roche) was used to detect apoptotic cells according to the manufacturer’s instruction. The cells were harvested and resuspended in Annexin-V-FLUOS buffer. The stained cells were observed under fluorescence microscope.

### Antibodies

Anti-Caspase-3 antibody (#9662), anti-PARP antibody (#9542), anti-phospho-Chk1 (Ser296) antibody (#2349), anti-phospho-Chk1 (Ser317) antibody (#2344), anti-phospho-Chk1 (Ser345) antibody (#2348), anti-Chk1 antibody (#2345), anti-phospho-Chk2 (Thr68) antibody (#2661), anti-phospho-Chk2 (Ser516) antibody (#2669), anti-Chk2 antibody (#2662), anti-phospho-p53 (Ser15) antibody (#9284), and anti-phospho-p53 (Ser20) antibody (#9287) were obtained from Cell Signaling Technology; anti-p53 antibody (DO-1; sc-126), anti-PRODH antibody (sc-376401), and HRP-conjugated anti-rat antibody (sc-2032) were from Santa Cruz Biotechnology; anti-α-tubulin antibody (T9026) and anti-γ-tubulin antibody (T6557) were from Sigma Aldrich; anti-p21 antibody (K0081-3) and anti-ATM antibody (PM026) were from Medical and Biological Laboratories; HRP-conjugated anti-rabbit antibody (W4011) and HRP-conjugated anti-mouse antibody (W4021) were from Promega; anti-HA antibody (1867423) was from Roche; anti-DAO antibody (6844-1) was from Abcam.

### Immunoblot analysis

The cells were lysed in lysis buffer (1% Nonidet P-40, 50 mM Tris-HCl [pH 7.5], 5 mM EDTA, 150 mM NaCl, 20 mM NaF, 20 mM β-glycerophosphate, 10 μg/ml leupeptin, 10 μg/ml aprotinin, 1 mM phenylmethanesulfonyl fluoride). The lysates were separated by SDS-PAGE and blotted onto Immobilon polyvinylidene difluoride membrane (Merck Millipore). Each protein was detected using primary antibodies, HRP-conjugated secondary antibodies, and the ECL detection reagent (GE Healthcare). Phos-tag SDS-PAGE was performed with polyacrylamide gels containing 100 μM Phos-tag (Wako) and 200 μM MnCl_2_.

### RNA interference

siRNAs for *ATM* (ATM_1: Hs_ATM_8 SI00604730, ATM_2: Hs_ATM_12 SI02663360, ATM_3: Hs_ATM_5 SI00299299, and ATM_4: Hs_ATM_9 SI00604737), *Chk1* (Chk1_1: Hs_CHEK1_8 SI00605094, Chk1_2: Hs_CHEK1_9 SI00287658, Chk1_3: Hs_CHEK1_7 SI00299859, and Chk1_4: Hs_CHEK1_13 SI02660007), *p53* (p53_1: Hs_TP53_3 SI00011655, p53_2: Hs_TP53_7 SI02623747, p53_3: Hs_TP53_9 SI02655170, and p53_4: Hs_TP53_13 SI04384079), and control siRNA (SI03650318) were obtained from Qiagen. HepG2 or U2OS cells were seeded and transfected with 30 nM siRNAs using HiPerFect Transfection Reagent (Qiagen) according to the manufacturer’s instruction.

### Plasmid constructions

The human *Chk1* cDNA (NCBI accession number NM_001114122.2) was amplified from plasmid pF1K-Chk1 (Promega; FXC03424) and cloned into downstream of the Flag tag sequence in the pcDNA3-Flag vector (Invitrogen) to generate pcDNA3-Flag-Chk1. The human *p21* cDNA (NM_001291549.1) was amplified from Flag-p21-WT (Addgene plasmid # 16240; a gift from Dr. M. C. Hung)^48^. The cDNA fragments of human *PVRL4* (NM_030916.2), *PRODH* (NM_016335.4), *LY6D* (NM_003695.2), *DAO* (NM_001917.4), and *EPN3* (NM_017957.2) were amplified from a cDNA sample prepared from U2OS cells. The cDNA fragment of human *GPR172B* (NM_001104577.1) was amplified from a plasmid containing *PAR2 (GPR172B*) cDNA (kindly provided by Dr. Y. Takeuchi). The resultant fragments were digested with appropriate restriction enzymes and cloned into downstream of the HA tag sequence in the pcDNA3-HA vector. To generate Chk1 mutant (S345A), PCR was performed using mutagenic primers. The sequences of primers used for plasmid constructions are listed in Supplementary Table 1.

### RNA isolation and microarray analysis

RNA was isolated using NucleoSpin RNA II (TaKaRa) according to the manufacturer’s instruction. The quantity and purity of the extracted RNA were evaluated using a NanoDrop ND-1000 spectrophotometer (Thermo Scientific). Expression profiling was performed by DNA Chip Research. The purified RNA was amplified and labeled with Cy3 using the One Color Low RNA Input Linear Amplification Kit (Agilent Technologies), then hybridized to Whole Human Genome DNA Microarray slides (4 × 44 K; Agilent Technologies), using the Agilent one-color gene expression hybridization protocol. Microarray slides were scanned in an Agilent Technologies G2505C Microarray Scanner at 5 μm resolution, and the scanned images were analyzed with Feature Extraction Software 10.7.3.1 (Agilent) using default parameters. The microarray analysis was carried out with a single sample, and the obtained data was used to select candidate genes for further analyses, based on the threshold cut-off criteria as described in the Results section. The microarray data have been deposited into the NCBI Gene Expression Omnibus (GEO) database (accession no. GSE61110).

### qPCR

cDNAs were generated from isolated RNA using ReverTra Ace qPCR RT Master Mix with gDNA Remover (TOYOBO). qPCR was performed using THUNDERBIRD SYBR qPCR Mix (TOYOBO) with the LightCycler480 Real-Time PCR System (Roche Applied Science). Primer sequences for qPCR are listed in Supplementary Table 1. Relative expression levels were quantified by constructing a standard curve using serial dilutions of the cDNA samples. *GAPDH* was served as endogenous normalization control.

### ChIP

ChIP assay was performed using the EZ Magna ChIP kit (Merck Millipore). Ten million cells were used per each ChIP experiment. The cells were cross-linked with 1% formaldehyde, treated with 125 mM glycine, and then lysed and sonicated with a Bioruptor UCD-300 (Cosmo Bio) for 4 min at high power. Antibody incubation with chromatin was performed overnight at 4 °C with 1 μg of p53 antibody (DO-1). Antibody-chromatin complexes were washed four times with Wash Buffers and eluted with ChIP Elution Buffer. Cross-links were reversed by incubation at 95 °C for 10 min, and DNA was purified with Spin column. Purified DNA was quantified by qPCR as described above. The JASPAR database (http://jaspar.binf.ku.dk) was used to search for putative p53 response elements. Primer sequences are listed in Supplementary Table 1.

### Statistical analysis

The unpaired, two-tailed Student’s *t*-test was used to calculate *P*-values for all datasets.

## Additional Information

**How to cite this article**: Nagano, T. *et al*. Identification of cellular senescence-specific genes by comparative transcriptomics. *Sci. Rep.* **6**, 31758; doi: 10.1038/srep31758 (2016).

## Supplementary Material

Supplementary Information

## Figures and Tables

**Table 1:** Relative mRNA levels of selected genes in HepG2 cells treated with 10 and 100 μM etoposide for 30 h.

Gene	Microarray results (Fold change)			qPCR results (Fold change ± SD)		Molecular class
	10 μM/DMSO	100 μM/DMSO	10 μM/100 μM	10 μM/DMSO	100 μM/DMSO	
***PVRL4***	32.09	14.46	2.22	22.30 ± 0.74	15.37 ± 0.65	Adhesion molecule
***GPR172B***	19.25	9.57	2.01	45.13 ± 1.24	23.71 ± 1.39	G protein coupled receptor
***DAO***	13.55	6.62	2.05	15.69 ± 2.62	9.46 ± 0.93	Oxidase
*CCDC74B*	9.47	2.81	3.37	1.56 ± 0.13	1.58 ± 0.10	Unclassified
***LOXL4***	8.66	3.39	2.55	5.41 ± 0.32	2.95 ± 0.38	Oxidase
***EVL***	8.65	4.11	2.10	16.36 ± 1.39	3.65 ± 0.08	Cytoskeletal protein
***PRODH***	8.42	3.71	2.27	8.62 ± 0.57	3.66 ± 0.46	Dehydrogenase
***E2F7***	7.94	1.21	6.56	7.59 ± 0.74	1.63 ± 0.02	Transcription regulation
***LY6D***	7.45	1.58	4.72	6.66 ± 0.23	1.80 ± 0.06	Adhesion molecule
***IGFBP2***	7.30	3.48	2.10	33.60 ± 1.54	10.75 ± 0.23	Secreted polypeptide
*CRABP2*	7.16	1.89	3.79	1.11 ± 0.14	1.44 ± 0.16	Transcription regulation
***EPN3***	6.41	3.07	2.09	6.98 ± 0.47	3.17 ± 0.32	Unclassified
***APOBEC3B***	4.99	1.22	4.09	5.96 ± 0.24	1.57 ± 0.16	RNA binding protein
***IER5***	3.85	1.80	2.14	3.66 ± 0.20	1.97 ± 0.05	Transcription regulation
***ANGPTL2***	3.63	1.76	2.06	3.00 ± 0.38	1.88 ± 0.13	Secreted polypeptide
***SLC48A1***	3.50	1.37	2.55	3.00 ± 0.11	1.61 ± 0.04	Transport/cargo protein
***WBSCR27***	3.41	1.61	2.12	2.94 ± 0.07	1.79 ± 0.35	Unclassified
***E2F2***	3.33	0.27	12.33	2.33 ± 0.15	0.33 ± 0.09	Transcription regulation
*NXPH4*	3.09	1.54	2.01	1.11 ± 1.03	1.06 ± 0.26	Secreted polypeptide
***PPM1D***	3.08	1.47	2.10	3.14 ± 0.13	1.80 ± 0.09	Serine/threonine phosphatase
*p21*	7.94	7.07	1.12	6.40 ± 0.54	5.43 ± 0.27	Cell Cycle control
*BTG2*	10.13	6.82	1.49	7.92 ± 0.81	7.17 ± 0.68	Transcription regulation
*SULF2*	19.37	9.78	1.98	10.60 ± 0.08	6.87 ± 0.09	Sulfatase

Twenty genes that were differentially upregulated at the low dose of etoposide were selected by microarray analysis. Differential upregulation of genes in bold type were confirmed by qPCR.

**Table 2:** Relative mRNA levels of identified genes in U2OS and SaOS-2 cells treated with 2 μM etoposide (Eto) and bleomycin (BLM) for 7 days.

Gene	Fold change ± SD			
	U2OS cells		SaOS-2 cells	
	Eto/DMSO	BLM/DMSO	Eto/DMSO	BLM/DMSO
*PVRL4*	8.27 ± 0.18	9.36 ± 0.48	1.77 ± 0.35	3.16 ± 0.07
*PRODH*	4.59 ± 0.16	6.85 ± 0.46	0.17 ± 0.09	0.55 ± 0.16
*LY6D*	4.10 ± 1.34	4.92 ± 0.84	0.57 ± 0.78	3.39 ± 2.07
*DAO*	3.92 ± 0.28	3.93 ± 0.62	0.55 ± 0.35	1.63 ± 0.09
*EPN3*	3.83 ± 0.46	4.39 ± 0.85	0.14 ± 0.07	0.17 ± 0.06
*p21*	3.47 ± 0.06	3.69 ± 0.01	1.13 ± 0.02	1.28 ± 0.02
*GPR172B*	2.53 ± 0.03	3.04 ± 0.05	0.83 ± 0.15	1.57 ± 0.08
*IGFBP2*	2.48 ± 0.60	2.20 ± 0.29	2.88 ± 0.33	2.32 ± 0.41
*E2F7*	1.92 ± 0.20	1.33 ± 0.03	1.77 ± 0.07	1.38 ± 0.08
*PPM1D*	1.39 ± 0.01	1.53 ± 0.04	0.70 ± 0.00	0.80 ± 0.02
*SLC48A1*	1.37 ± 0.07	1.06 ± 0.03	1.13 ± 0.09	1.33 ± 0.05
*APOBEC3B*	1.00 ± 0.01	1.44 ± 0.07	1.30 ± 0.11	1.26 ± 0.02
*IER5*	0.85 ± 0.02	0.74 ± 0.01	1.53 ± 0.02	1.51 ± 0.05
*ANGPTL2*	0.68 ± 0.02	0.67 ± 0.02	0.28 ± 0.02	0.50 ± 0.00
*E2F2*	0.24 ± 0.08	0.24 ± 0.03	1.32 ± 0.03	0.87 ± 0.11
*LOXL4*	0.14 ± 0.01	0.18 ± 0.01	0.81 ± 0.02	1.57 ± 0.05
*EVL*	N/D	N/D	N/D	N/D
*WBSCR27*	N/D	N/D	N/D	N/D

The levels of *p21* served as a marker for p53-dependent DNA damage responses. N/D, not detected.

**Table 3:** List of the identified genes.

Gene	Full name	Molecular class
*PVRL4*	poliovirus receptor-related 4	Adhesion molecule
*PRODH*	proline dehydrogenase (oxidase) 1	Dehydrogenase
*LY6D*	lymphocyte antigen 6 complex, locus D	Adhesion molecule
*DAO*	D-amino-acid oxidase	Oxidase
*EPN3*	epsin 3	Unclassified
*GPR172B*	solute carrier family 52, (riboflavin transporter), member 1	G protein coupled receptor
