# Supplementary material for: Identification of cellular senescence-specific genes by comparative transcriptomics
Source: Sci Rep. 2016 Aug 22;6:31758. doi: 10.1038/srep31758 (PMC4992837; doi:10.1038/srep31758)
Supplement: Supplementary Information [file srep31758-s1.pdf]

Supplementary Information for

Identification of cellular senescence-specific genes by comparative transcriptomics

Taiki Nagano[1,2], Masayuki Nakano[1,2], Akio Nakashima[1,3], Kengo Onishi[1,2], Shunsuke Yamao[1,2], Masato Enari[4], Ushio Kikkawa[1,3] & Shinji Kamada[1,2]*

[1]Biosignal Research Center, Kobe University, 1-1 Rokkodai-cho, Nada-ku, Kobe 657-8501, Japan.

[2]Department of Biology, Graduate School of Science, Kobe University, 1-1 Rokkodai-cho, Nada-ku, Kobe 657-8501, Japan.

[3]Department of Bioresource Science, Graduate School of Agricultural Science, Kobe University, 1-1 Rokkodai-cho, Nada-ku, Kobe 657-8501, Japan.

[4]Division of Refractory and Advanced Cancer, National Cancer Center Research Institute, 5-1-1 Tsukiji, Chuo-ku, Tokyo 104-0045, Japan.

*To whom correspondence should be addressed.

Email: skamada@kobe-u.ac.jp

Supplementary Table 1. List of primers used for plasmid constructions, qPCR, and ChIP-qPCR.

Gene	Forward	Reverse
(For plasmid constructions)		
Chk1 (Wt)	gctaacgaattcatggcagtgccctttgtgga	cgagcattgcggccgctcatgtggcaggaagccaaatct
Chk1 (S345A)	gtacaagggatcagctttgctcagcccacatgtcctgat	atcaggacatgtgggctgagcaaagctgatcccttgtac
p21	gcgaattcaccatgtcagaaccggctgggg	cgttaatagcggccgcttagggcttcctcttggagaa
PVRL4	cgtggatcgaattcatgcccctgtccctgggagccgagat	cgtgctcggcggccgctcagaccaggtgtccccgcccatt
PRODH	gcgaattcaccatggctctgaggcgcgc	cgtctcgagctaggcagggcgatggaag
LY6D	cgtgctcggaattcatgaggacagcattgctgct	cgtgctcggcggccgctcacaggctgggggctaaga
DAO	cgtgctcggaattcatgcgtgtggtggtgattggag	cgtgctcggcggccgctcagaggtgggatggtggcatt
EPN3	gtatctatgaattcatgacgacctccgcactccg	gtatctatgcggccgctcagaggaaggggttggtgcc
GPR172B	cgtgctcggaattcatggcagcacccacgct	cgtgataggcggccgctcaggggccacaggggt
(For qPCR)		
GAPDH	caatgaccccttcattgacct	atgacaagcttcccgttctc
PVRL4	ctgccatgtcagcaatgagt	tcctgggggtcaagaacat
LY6D	tggaatgctgatgacttggag	acagaaggagtgtgaaatccg
GPR172B	ttgctgttgccatcactacc	caaagcctcttcttcctccttc
DAO	ccactggacataaaggtctacg	gggttgttggggtcagaaag
LOXL4	ccaaagactggacgcgata	aggaggtcgtagtgggtgaa
EVL	gcctgtgtcctcgattctgt	tcttagcttcggggctcttt
PRODH	catcgaagcctcaggtagagt	ccccagtgctgtgagcttaat
E2F7	tccagattcccaggaacaac	tggttttggagacgaggaac
CRABP2	tgcgcaccacagagattaac	cccatttcaccaggctctta
IER5	ttacagacagaagcccgaagt	tcagctcccccaacctttat
E2F2	tgaaggagctgatgaacacg	agcacggatatcctggtaagtc

Supplementary Table 1. List of primers used for plasmid constructions, qPCR, and ChIP-qPCR (Continued).

Gene	Forward	Reverse
(For qPCR)		
IGFBP2	tgcaagatgtctctgaacgg	cattgtagaagagatgacactcgg
ANGPTL2	acagcacaaagaacaactcctc	ttcggaaaacagaatccagc
NXPH4	ctttccccgcagctttaat	ggacaggccgaggtttttag
APOBEC3B	cctggttccttctttgcagtt	tgtgttctcctgaaggaacg
SLC48A1	ggcagttctcgagctatctgatta	ggcacaactgaactagcgga
PPM1D	ggaggtgacacaggaccataa	cgattcaccccagacttgttc
CCDC74B	gctttgagctacccgcat	caggcctaaaagtagcggaagt
EPN3	cttggctgacatcttcgtacct	tgtgttcggcctaaaacctg
WBSCR27	tgatagtcggtgccctcagt	ccttgtattgaaggttggacg
p21	cgactgtgatgcgctaatg	tctcggtgacaaagtcgaag
BTG2	agcctcatggtctcatgctta	cagctcaggggttttgttg
SULF2	cgaccacggttaccacatc	ggaccctgatgtcaaactcata
(For ChIP-qPCR)		
GAPDH (-32 to +134)	tactagcggttttacgggcg	tcgaacaggaggagcagagagcga
PVRL4 (-1563 to -1467)	cttcaccaaaatgtagtcagttcc	ctccttgaaagttgggcttgt
PRODH (+6823 to +6904)	ttgcctcagcatgtcgg	caaaacagccaatcgcaag
LY6D (-2776 to -2626)	tgagcaaggaacttcggc	cagaaatgacacctgggagag
DAO (+2144 to +2540)	cagtgaggagacaataaggcaa	gagttggttaatcctaatcgaacct
EPN3 (-2038 to -1948)	caacttgtctgggcttgtatga	aggccaattttccttcctgt
GPR172B (+2112 to +2250)	atgctctttggggcctacc	cacttgctccaggggacac
p21 (-40 to +65)	gtggctctgattggctttctg	ctgaaaacaggcagcccaag

The exact positions of primers used to amplify the respective genes in ChIP-qPCR are given as nucleotide distance from transcription start sites in parentheses.

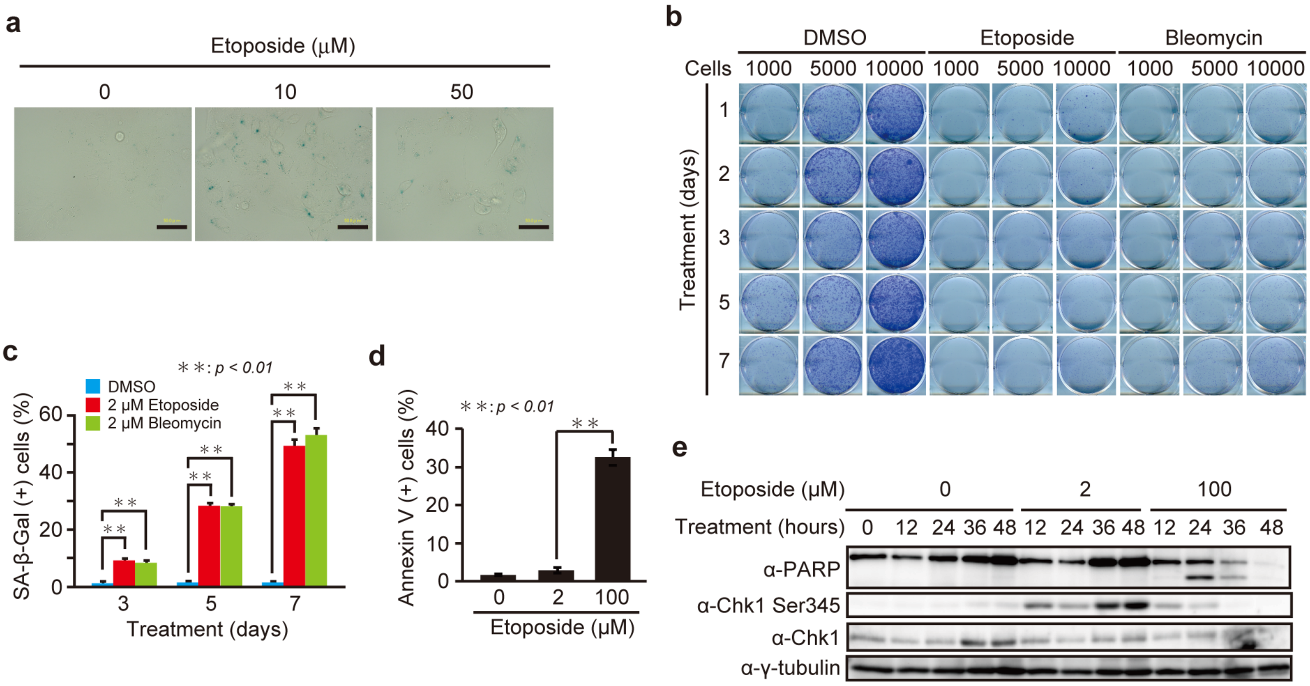

Supplementary Figure 1. Senescence is induced by the low dose of etoposide, whereas apoptosis is triggered at higher doses. (**a**) HepG2 cells treated with 10 and 50 μM etoposide for 48 h were subjected to SA-β-Gal staining. Representative images are shown. Bars, 50 μm. (**b**) U2OS cells were treated with 2 μM etoposide and bleomycin for 1, 2, 3, 5, and 7 days, and then replated in 35-mm dish at 1000, 5000, and 10000 cells per well. The cells were cultured in the medium without drugs for 10 days and stained with crystal violet. (**c**) U2OS cells were treated with 2 μM etoposide and bleomycin for 3, 5, and 7 days, and subjected to SA-β-Gal staining. The percentage of SA-β-Gal-positive cells was quantified. (**d**) U2OS cells were treated with 2 and 100 μM etoposide for 48 h and subjected to Annexin V

staining. The percentage of Annexin V-positive cells was quantified. (e) Lysates from U2OS cells treated with 2 and 100 μM etoposide for 12, 24, 36, and 48 h were subjected to immunoblot analysis. Data are mean ± SD. Statistical significance is shown using the Student's *t*-test analysis ($n = 3$); $^{**}P < 0.01$.

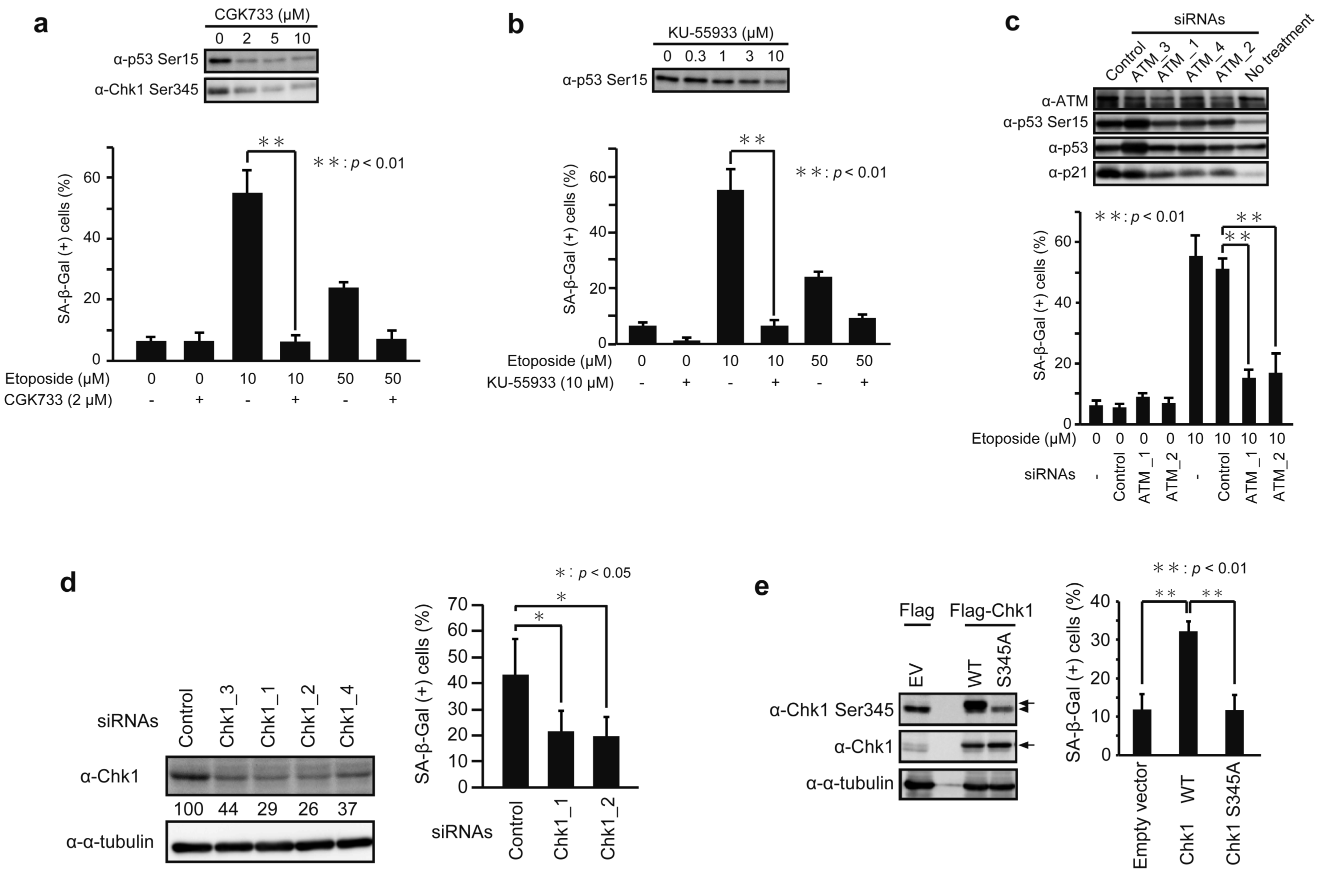

Supplementary Figure 2. Inhibition or depletion of DDR components prevents DNA damage-induced senescence. **(a,b)** (Upper) HepG2 cells pretreated with CGK733, an ATM/ATR inhibitor (**a**), and KU-55933, an ATM inhibitor (**b**) at indicated doses for 1 h were treated with 10 μM etoposide for 48 h, and then subjected to immunoblot analysis. Drug inhibition of ATM/ATR was shown by abrogation of p53 phosphorylation at Ser15 (the ATM target site) and Chk1 phosphorylation at Ser345 (the ATR target site). (Lower) HepG2 cells pretreated with 2 μM CGK733 (**a**) and 10 μM KU-55933 (**b**) for 1 h were then treated with 10 and

50 μM etoposide for 48 h and subjected to SA-β-Gal staining. The percentage of SA-β-Gal-positive cells was quantified. (**c**) HepG2 cells were transfected with siRNAs for negative control (Control) and *ATM* (ATM_1, ATM_2, ATM_3, and ATM_4). After incubation for 48 h, the cells were treated with 10 μM etoposide for 48 h, and were subjected to immunoblot analysis (upper) and the percentage of SA-β-Gal-positive cells was quantified (lower). (**d**) (Left) U2OS cells were transfected with siRNAs for negative control (Control) and *Chk1* (Chk1_1, Chk1_2, Chk1_3, and Chk1_4). After incubation for 48 h, Chk1 expression was determined by immunoblot analysis. The Chk1 protein level relative to the α-tubulin level was quantified using NIH ImageJ software and is indicated at the bottom of each lane. (Right) *Chk1*-depleted cells were treated with 2 μM bleomycin for 7 days and subjected to SA-β-Gal staining. (**e**) (Left) U2OS cells transfected with pcDNA3-Flag containing WT and S345A Chk1 were selected with 800 μg/ml G418 for 5 days. The cells were then treated with 5 μM etoposide for 48 h and subjected to immunoblot analysis. Arrowhead and arrows indicate endogenous and exogenous Chk1, respectively. (Right) U2OS cells overexpressing WT or S345A Chk1 were treated with 2 μM etoposide for 7 days and subjected to SA-β-Gal staining. Data are mean ± SD. Statistical significance is shown using the Student's *t*-test analysis (n = 3); *P < 0.05; **P < 0.01.

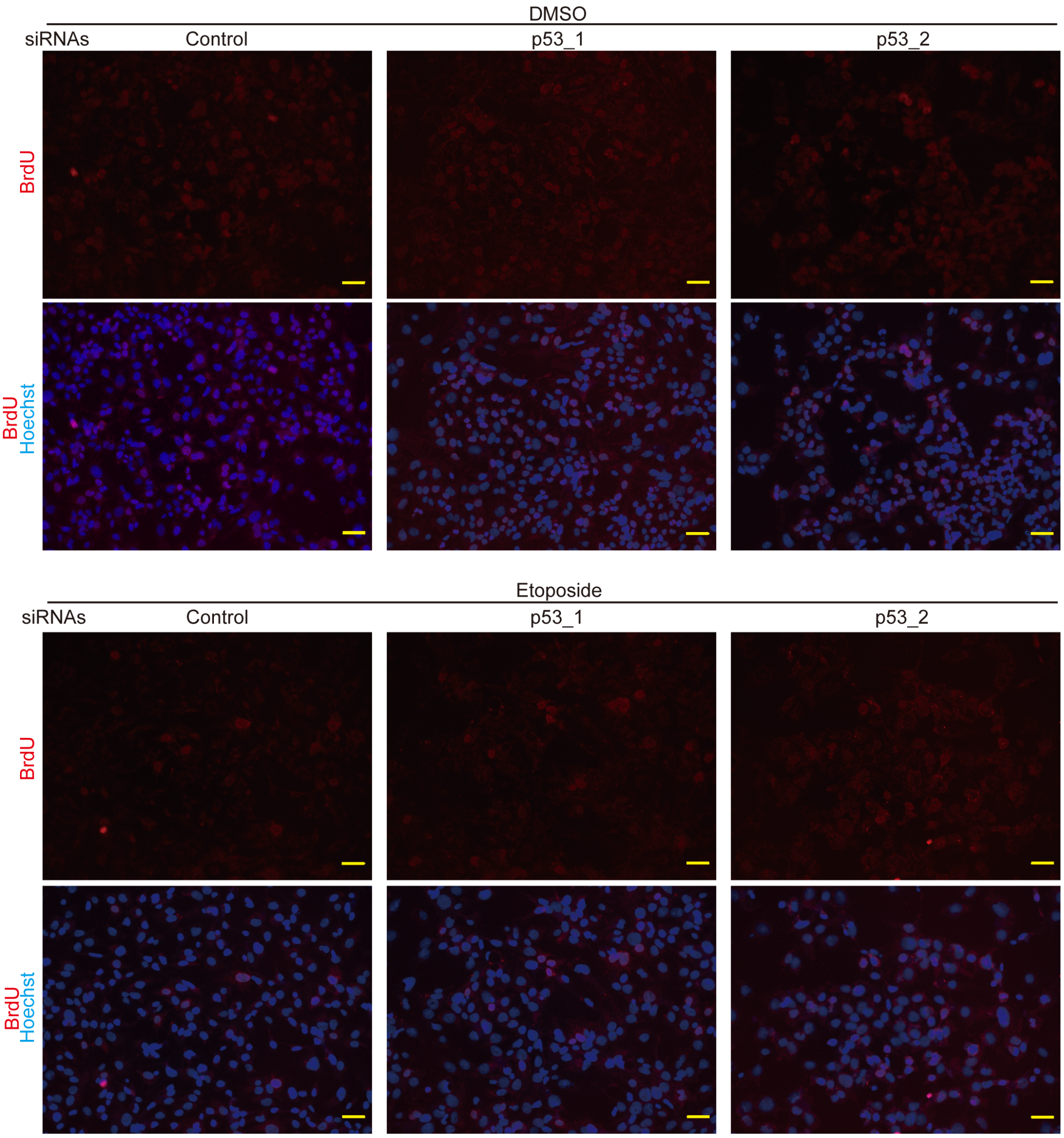

Supplementary Figure 3. Etoposide-induced loss of proliferative capacity is reversed by *p53* knockdown. HepG2 cells transfected with siRNAs for *p53* (p53_1 and p53_2) were treated with 10 μM etoposide for 48 h and subjected to BrdU incorporation assay. Representative images are shown. Bars, 50 μm.

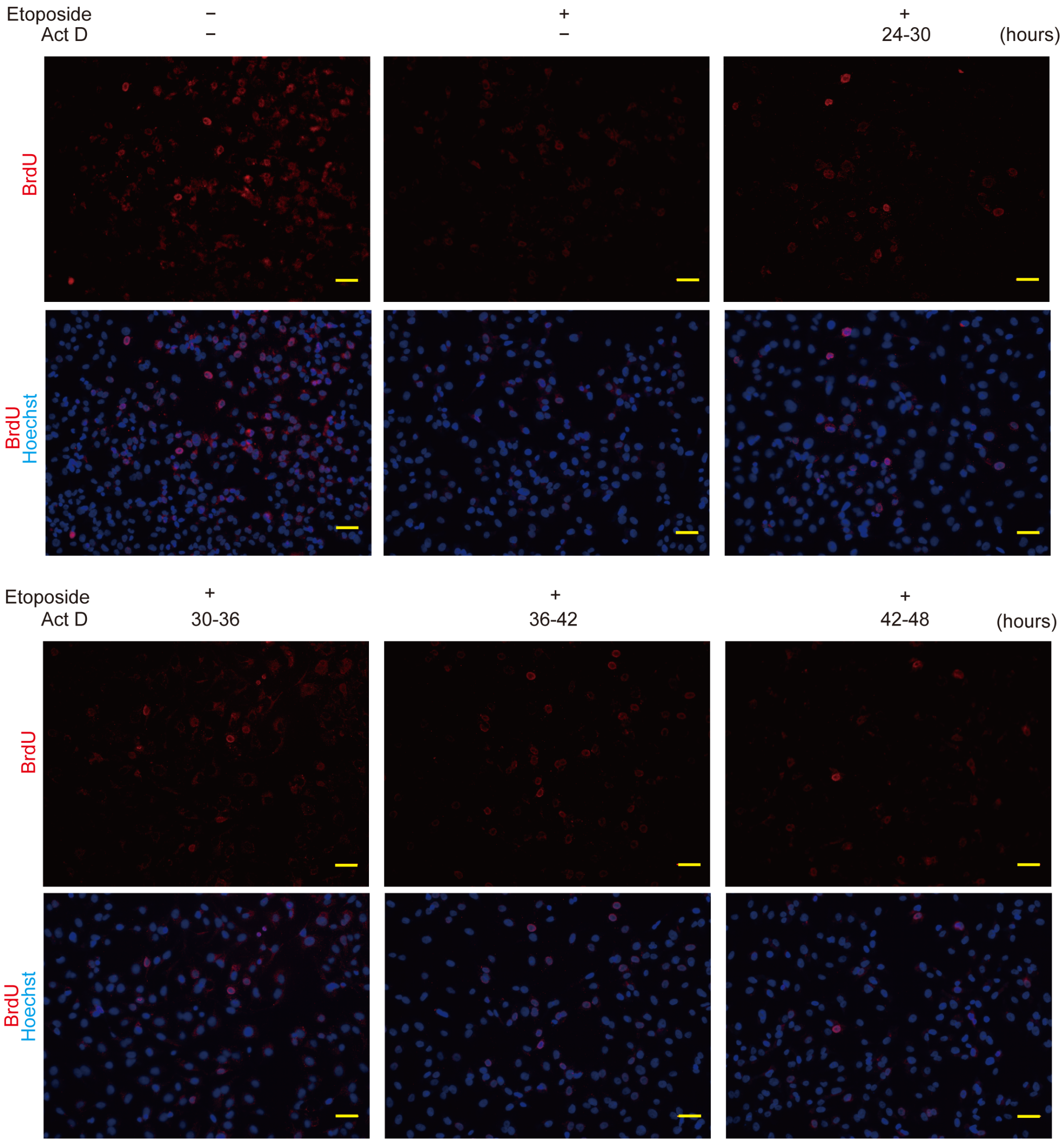

Supplementary Figure 4. Etoposide-induced loss of proliferative capacity is reversed by treatment with Act D. HepG2 cells were treated with 10 μM etoposide for various times (24, 30, 36, and 42 h), and then Act D was added to the medium at a concentration of 50 ng/ml. After 6 h of incubation in the presence of Act D and etoposide, the drugs were washed out by replacing the medium, and the cells retreated only with

etoposide up to for 48 h after initial exposure to etoposide were subjected to BrdU incorporation assay. Representative images are shown. Bars, 50 μm.

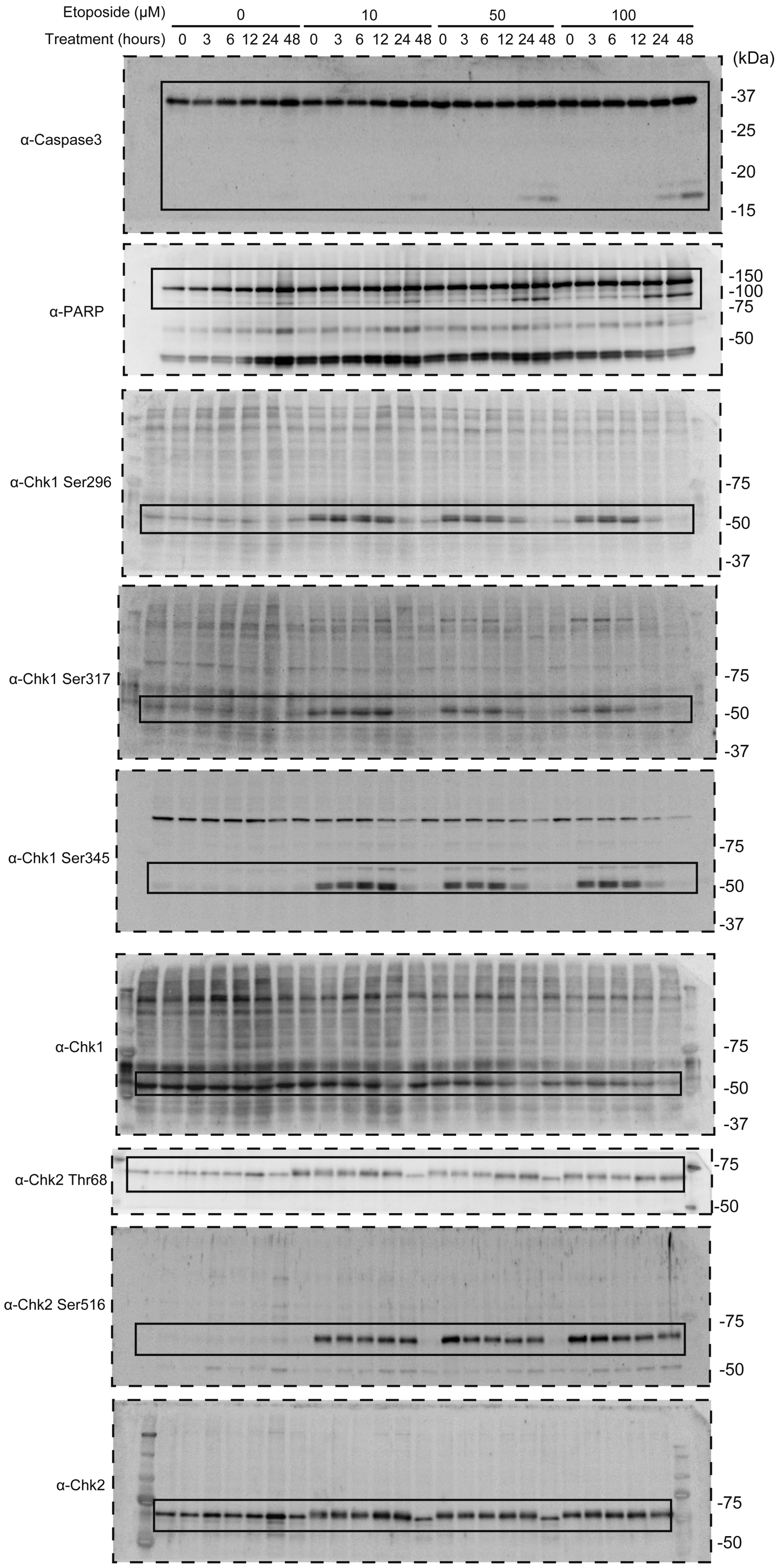

Supplementary Figure 5. Full-length immunoblot images of Figure 1.

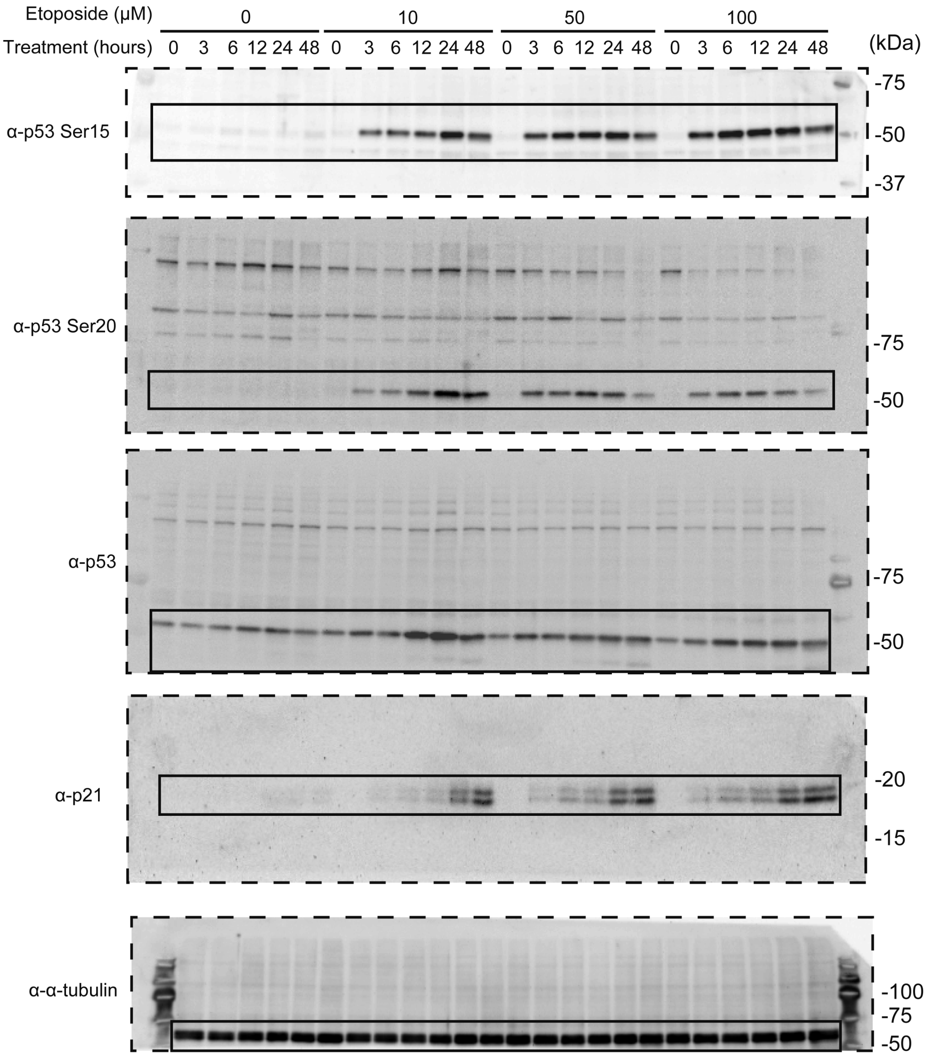

Supplementary Figure 5. Full-length immunoblot images of Figure 1 (Continued). Uncropped images of blots in Figure 1d are shown. Cropping lines are indicated with squares. The dotted lines indicate the margins of the gels.

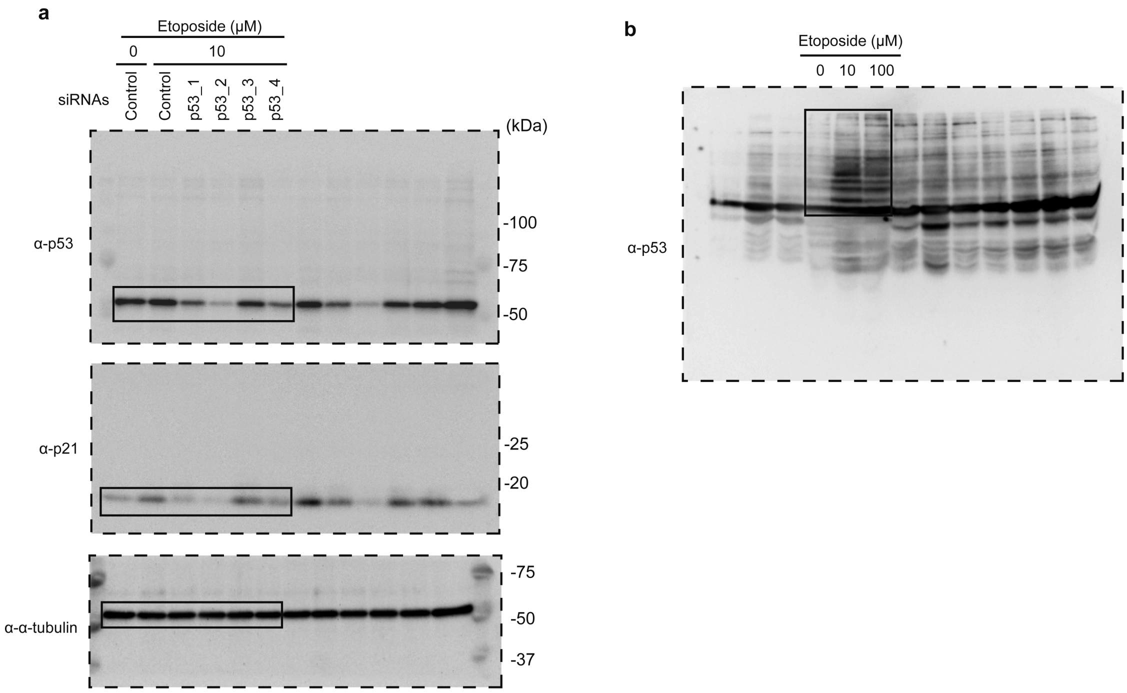

Supplementary Figure 6. Full-length immunoblot images of Figure 2. (**a**,**b**) Uncropped images of blots in Figure 2a (**a**) and f (**b**) are shown. Cropping lines are indicated with squares. The dotted lines indicate the margins of the gels.

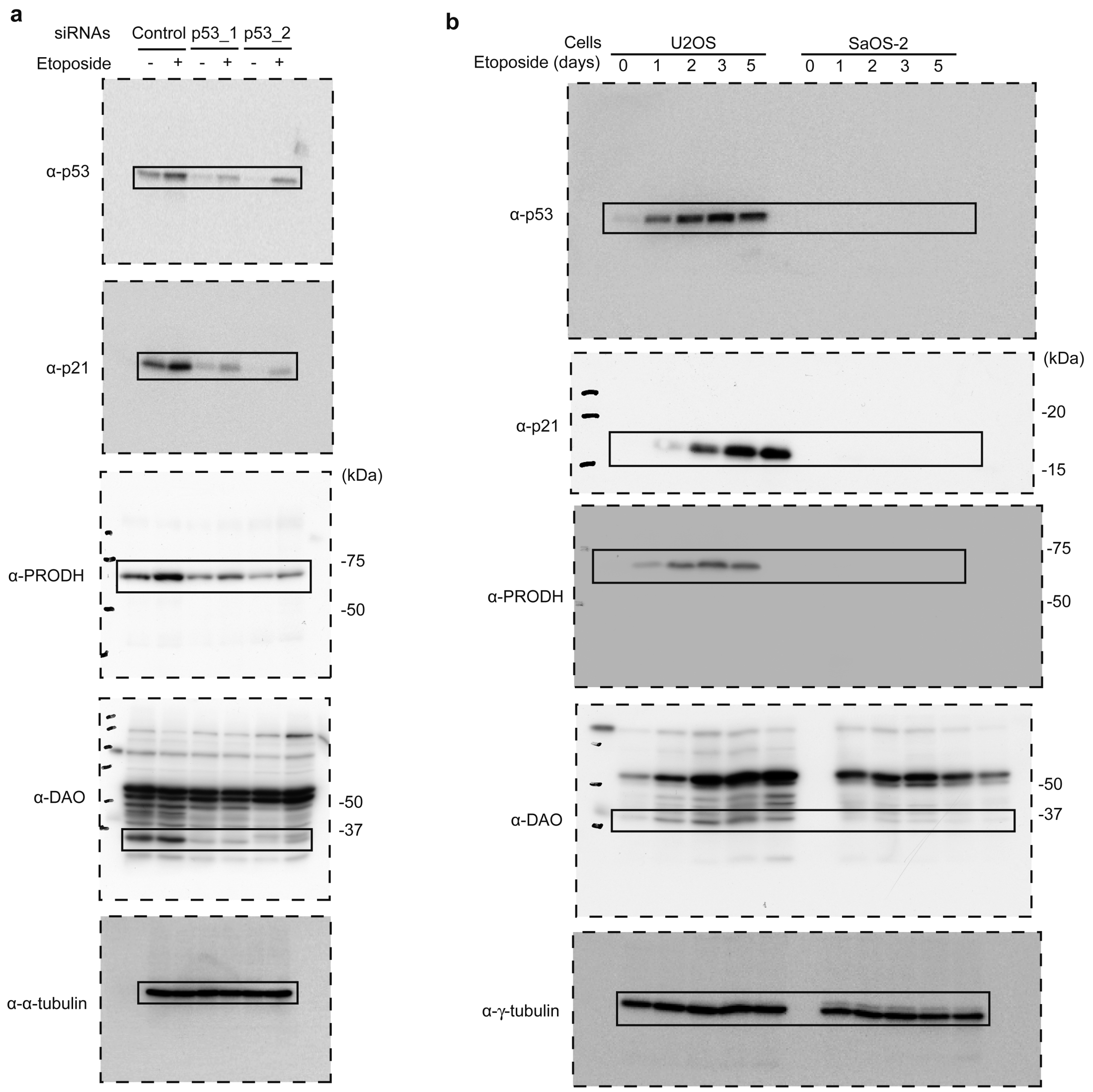

Supplementary Figure 7. Full-length immunoblot images of Figure 3. (**a**,**b**) Uncropped images of blots in Figure 3c (**a**) and d (**b**) are shown. Cropping lines are indicated with squares. The dotted lines indicate the margins of the gels.

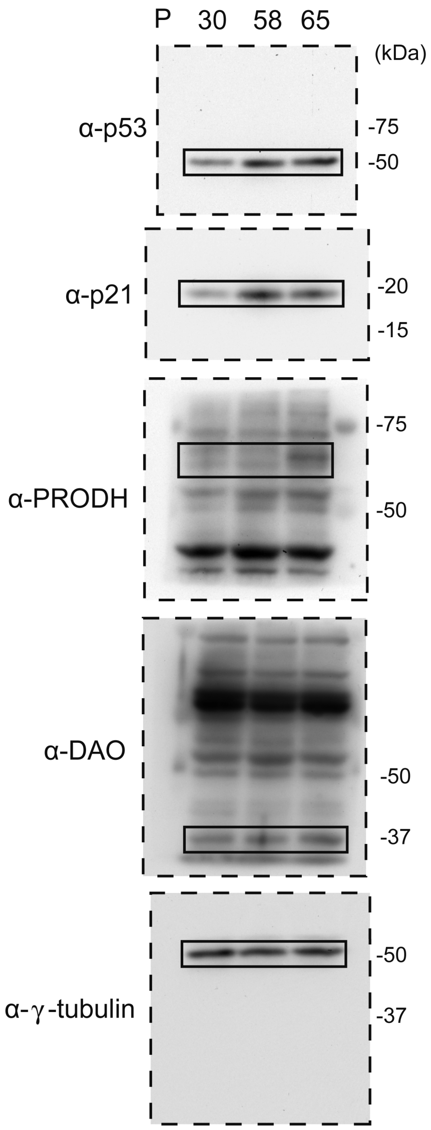

Supplementary Figure 8. Full-length immunoblot images of Figure 4. Uncropped images of blots in Figure 4c are shown. Cropping lines are indicated with squares. The dotted lines indicate the margins of the gels.

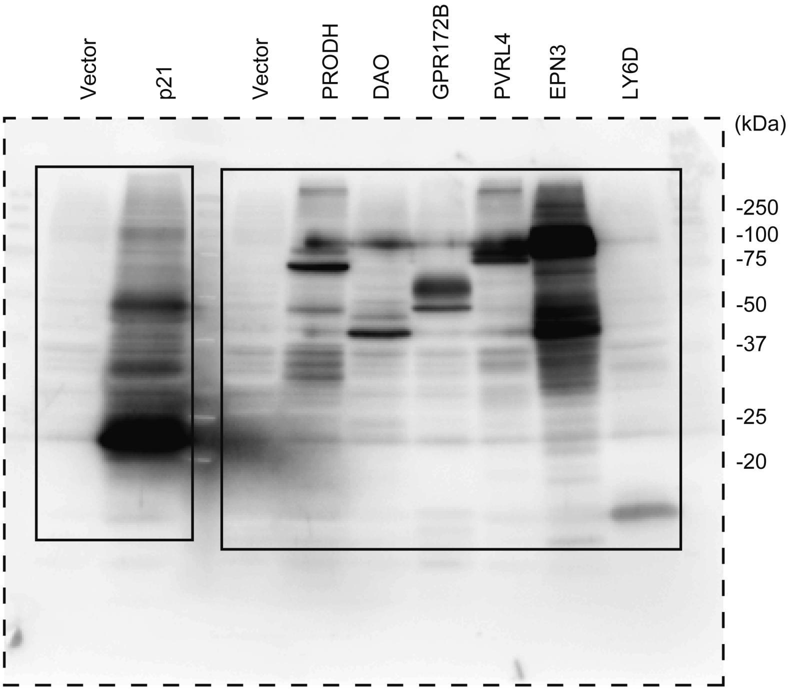

Supplementary Figure 9. Full-length immunoblot image of Figure 5. Uncropped image of the blot in Figure 5a is shown. Cropping lines are indicated with squares. The dotted line indicates the margin of the gel.
